# Knowledge, attitudes and practices regarding malaria prevention and control in communities in the Eastern Region, Ghana, 2020

**DOI:** 10.1371/journal.pone.0290822

**Published:** 2023-08-30

**Authors:** Aquel Rene Lopez, Charles Addoquaye Brown

**Affiliations:** 1 Tetteh Quarshie Memorial Hospital, Mampong, Ghana; 2 Department of Medical Laboratory Sciences, School of Biomedical and Allied Health Sciences, College of Health Sciences, University of Ghana, Korle Bu, Accra, Ghana; University of Health and Allied Sciences, GHANA

## Abstract

**Background:**

In sub-Saharan Africa countries including Ghana, the malaria burden remains unacceptably high and still a serious health challenge. Evaluating a community’s level of knowledge, attitude, and practice (KAP) regarding malaria is essential to enabling appropriate preventive and control measures. This study aimed to evaluate knowledge of malaria, attitudes toward the disease, and adoption of control and prevention practices in some communities across the Eastern Region of Ghana.

**Methods:**

A cross‑sectional based study was carried out in 13 communities across 8 districts from January -June, 2020. Complete data on socio-demographic characteristics and KAP were obtained from 316 randomly selected household respondents by a structured pre-tested questionnaire. Associations between KAP scores and socio-demographic profiles were tested by Chi-square and binary logistic regression. Data analysis was done with SPSS version 26.0.

**Results:**

Most respondents (85.4%) had good knowledge score about malaria. Preferred choice of treatment seeking place (50.6%) was the health center/clinic. All respondents indicated they would seek treatment within 24 hours. Mosquito coils were the preferred choice (58.9%) against mosquito bites. Majority of households (58.5%) had no bed nets and bed net usage was poor (10.1%). Nearly half of the respondents (49.4%) had a positive attitude toward malaria and 40.5% showed good practices. Chi-square analysis showed significant associations for gender and attitude scores (p = 0.033), and educational status and practice scores (p = 0.023). Binary logistic regression analysis showed that 51–60 year-olds were less likely to have good knowledge (OR = 0.20, p = 0.04) than 15–20 year-olds. Respondents with complete basic schooling were less likely to have good knowledge (OR = 0.33, p = 0.04) than those with no formal schooling. A positive attitude was less likely in men (OR = 0.61, p = 0.04). Good malaria prevention practice was lower (OR = 0.30, p = 0.01) in participants with incomplete basic school education compared to those with no formal schooling.

**Conclusion:**

Overall scores for respondents’ knowledge, though good, was not reflected in attitudes and levels of practice regarding malaria control and prevention. Behavioral change communication, preferably on radio, should be aimed at attitudes and practice toward the disease.

## Introduction

In sub-Saharan Africa, the malaria burden remains unacceptably high and still a serious health challenge [1]. The West African sub-region, due to its current high rates of malaria infections and fatalities, is a hotspot for the disease’s transmission. The sub-region accounts for about half of the malaria global burden [1, 2].

Ghana is among the countries in West Africa with the highest burden of malaria [3]. Ghana has a hyperendemic malaria problem, thus every region is susceptible to infection [4, 5]. The most vulnerable populations are children under 5 years and pregnant women, with malaria accounting for 40.0% of all outpatient attendance [6].

One of the seven work packages created by the WHO Strategic Advisory Group on Malaria Eradication (SAGme) is community engagement for the elimination and eradication of malaria [7]. Thus, the necessity of inclusive and cooperative efforts to eradicate malaria and the significance of maintaining the target community at the forefront of the fight against the disease cannot be overstated. The willingness of each community member to participate and act is greatly influenced by their attitudes toward the disease and the use of any existing control measures if a malaria control program is to succeed in reducing both morbidity and mortality in that community [8]. These attitudes are influenced by their level of knowledge, understanding, and perception [9, 10]. For example, decisions likely to be taken when putative malaria symptoms set in such as, whether to just stay at home, use herbal treatment, buy over-the-counter drugs for self -medication, or go to a health facility [9, 10] may affect treatment outcomes. “Fever”, which is synonymous with malaria in many settings, unfortunately is present in other tropical diseases common in malaria-endemic areas [11]. It thus is important that individuals experiencing a fever seek medical attention as soon as possible to rule out malaria or other possible causes of their illness [11]. Any choice that ultimately results in delayed diagnosis and treatment, particularly in children, may have fatal consequences [12, 13].

Knowledge, attitudes, and practices (KAP) surveys are frequently used tools that can gather crucial data to inform the design of malaria control and prevention activities and interventions, ensuring community participation, acceptance, and adherence [14, 15]. Malaria KAP surveys have been conducted in many African countries including Cape Verde [16], Cameroon [17, 18], Ethiopia [19–22], Nigeria [23, 24] and Senegal [25]. Similar studies have also been conducted in some regions in Ghana [26–29]. However, to our knowledge, there is a scarcity of data on malaria KAP in the Eastern Region of Ghana. The current study therefore aimed to evaluate knowledge of malaria, attitudes toward the disease, adoption of control and prevention practices, and care-seeking behaviors in communities across the Eastern Region of Ghana. The study also investigated variables linked to KAP regarding malaria.

## Methods

### Study area and population

**Fig 1:**
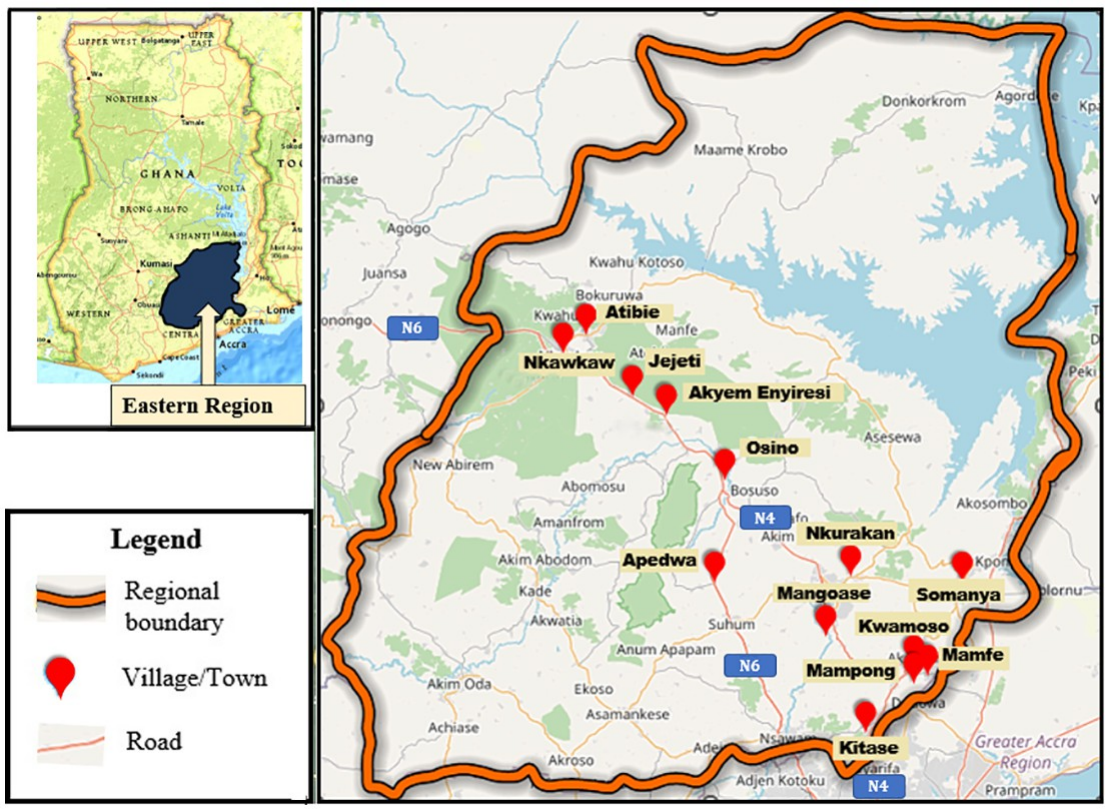
Map of the Eastern Region of Ghana (insert) showing the selected districts and communities. Ghana and Eastern Region maps adapted from USGS National Map Viewer.

The Eastern Region (Fig 1) has a land area of 19,323 square kilometers (which is 8.1% of the total land area of Ghana) [30]. Koforidua is the administrative capital. The Region can be described as urbanized (over 50% of the population live in urban areas). Based on the 2021 population census it has a population 2,917,039 which makes up 9.5% of the total population of the country [31]. There are 49.2% men and 50.8% women in the population, with an urban-rural divide of 43.3% to 56.6%, respectively. About 41.3% of the population is < 5 years. About 53% of the population are engaged in agriculture which is the region’s primary economic activity; 10.7% and about 22% of the population are in industry and in the services sub-sector, respectively.

### Study design and sampling strategy

This household-based cross-sectional descriptive and analytical survey was conducted in 13 communities across 8 districts in the Eastern Region of Ghana. The study was conducted between January -June, 2020.

For this KAP study, 8 districts accessible by the N6 and N4 highways were chosen for logistical feasibility. All districts also had ≤5 km intra-regional proximity to a healthcare facility. Communities in each district were chosen through systematic random sampling. The community was sampled for participants using a two-stage cluster sampling method. Six geographic divisions of the community were made, and four of them were chosen. Households were located within chosen clusters using proportional systematic random sampling to guarantee that every household had an equal chance of being included. Every third household was surveyed after a randomly chosen starting point (a household) was made. When a home was empty or the residents were away when the visit was made, the nearest household that had an eligible participant was contacted.

In the presence of the household head or the spouse, or an adult (over 18 years old) inhabitant, the goal of the study was explained and the willingness to participate in the study sort. Thus, the study’s participants were households/ household respondents that provided informed verbal consent. Only one individual per household was interviewed; either only the household head or the spouse, or adult inhabitant. The spouse or another family member who was an adult was questioned only when the household head was not available.

### Sample size

Sample size was calculated using the StatCalc Sample Size and Power function in Epi Info 7.2.5.0. Using a population size of 2,917,039, [31] an expected frequency of 70%, acceptable margin of error of 5%, design effect 1.0% and leaving both design effect and cluster equal to 1, a sample size of 323 was estimated at 95% confidence level.

### Ethical considerations

This study was approved by the Ethics and Protocol Review Committee of the School of Biomedical and Allied Health Sciences, College of Health Sciences, University of Ghana, Korle-bu, Accra. Verbal consent was also obtained from the household respondents before interview.

### Data collection (KAP surveys)

House-to-house surveys were conducted by adequately trained field staff. After gaining free and informed consent, the interviewee was given a structured, standardized, and pre-tested questionnaire translated into the Akan local language. The questionnaire comprised 58 questions, and was divided into six main sections. The first section collected information on socio-demographic characteristics. The second section addressed basic knowledge of malaria with a sub-section on bed net use and the third section, sources of information about malaria. The fourth and fifth sections addressed treatment seeking behaviors and attitudes toward malaria, respectively. The last section addressed practices toward malaria prevention. The questionnaire was adapted from the 2019 Malaria Indicator Survey in Ghana [32] and different peer reviewed KAP papers [20, 33, 34].

### Data analysis

Data collected was entered and analyzed using SPSS version 26.0 (IBM Corp, NY, USA). Relevant tables and figures were created from the data to allow for easy analysis and interpretation. Continuous variables such as age were presented in ranges. Categorical variables were reported using descriptive statistics such as frequencies and percentages. Variables analyzed included socio-demographic factors and malaria-related KAP.

These criteria were used to determine the participants’ knowledge scores regarding malaria. The responses to five different questions—including the mode of transmission, malaria signs and symptoms, and preventive measures—were combined to determine the respondents’ knowledge of malaria. Each correct knowledge response received a score of 1, while an incorrect or uncertain response received a score of 0. A modified Bloom’s cut-off was adapted using a score of above 60% of correct answers to indicate good knowledge and a score of below 60% to indicate poor knowledge [35, 36].

Attitude was measured using Likert scaling technique. According to the Likert scale, responses to the affirmative and negative statements ranged from strongly agree (score of 4), agree (score of 3), disagree (score of 2), and to strongly disagree (score of 1). Each respondent received a total score after the responses were added up. When the mean score was calculated, respondents with scores greater than or equal to 40.4 were considered to have a positive attitude toward malaria, while those with scores below 40.4 were considered to have a negative attitude.

Using the Likert scale, the respondents’ practices were also identified. The Likert scale scoring system was applied to the respondents’ responses, with scores ranging from never (score of 0), sometimes (score of 1), and always (score of 2). Each respondent’s total score was calculated after the responses were added up, and the study participants’ mean practice scores were calculated as well. When a respondent’s overall practice score was greater than the mean practice score (6.3), he/she was considered to have good practice. However, when an overall practice score fell below the mean practice score (6.3), that respondents was considered to have poor practice.

To examine the relationships between knowledge, attitude and practice scores and socio-demographic profile, Chi-square tests were carried out. Age, gender, and education were the independent variables. To determine how the independent variables associated with the dependent variable, binary logistic regression analyses were carried out. Statistical significance was defined at p < 0.05.

## Results

### Socio-demographic data

A total of 316 completed questionnaires of household respondents from 13 communities in 8 districts (Table 1) were used in the analysis of the survey data. The highest number of respondents (14.2%) were sampled in Apedwa, with the least (1.3%) from Somanya. Majority of the respondents were males (60.4%) (Table 2). Household heads (32.3%) and adult inhabitants not related to the household heads (21.2%) were the highest respondents. The age category of 41–50 years had the largest number of respondents (37.7%). Majority of the respondents had gone to school with 25.0% having completed primary school and 20.9% with post-secondary school qualifications. However, about a fifth (19.6%) of the respondents had no formal schooling. Household sizes of six or more (38.6%) and four or five (38.0%) were the highest. Only four (1.3%) respondents stayed alone. Majority of the houses in the communities had unburnt bricks, mud and poles, thatch/straw, timber, etc. as the major construction material of the house external wall (54.7%), and iron sheets or tiles for the major construction material for the house roof (88.0%). The main sources of income were agriculture based (38.3%) and craft/creative work (35.4%). All the respondents had a form of trading as a second source of income (Table 2).

**Table 1:** Number of households sampled in each community per district.

District	Community	Frequency	Percent (%)
Akuapim North Municipal	Kwamoso	20	6.3
	Mamfe	29	9.2
	Mampong	15	4.7
	Mangoase	24	7.6
Akuapim South	Kitase	6	1.9
Atiwa East	Akyem Enyiresi	39	12.3
	Jejeti	32	10.1
Abuakwa South Municipal	Apedwa	45	14.2
Fanteakwa South	Osino	37	11.7
Kwahu South	Atibie	29	9.2
Kwahu West	Nkawkaw	20	6.3
Yilo Krobo Municipal	Nkurakan	16	5.1
	Somanya	4	1.3
	Total	316	100

**Table 2:** Socio-demographic characteristics of respondents.

Variable	Categories	Frequency	Percent (%)
Gender of respondent	Female	125	39.6
	Male	191	60.4
What is your relationship to the head of the household?	Head of household	102	32.3
	Spouse/partner	32	10.1
	Son/daughter	39	12.3
	Grandchild	36	11.4
	Parent	36	11.4
	Brother /sister	4	1.3
	Not related	67	21.2
Age (years)	15–20	56	17.7
	21–30	74	23.4
	31–40	49	15.5
	41–50	119	37.7
	51–60	14	4.4
	Above 60	4	1.3
Highest level of education	No formal schooling	62	19.6
	Incomplete basic school	38	12.0
	Complete basic school	79	25.0
	Incomplete secondary school (SHS)	33	10.4
	Complete secondary school (SHS)	32	10.1
	Post-secondary e.g., certificate, diploma	66	20.9
	Degree and above	6	1.9
Number of persons in household, including respondent	Six or more	122	38.6
	Four or five	120	38.0
	Three	54	17.1
	Two	16	5.1
	One	4	1.3
Major construction material of the house roof	Thatch, straw, or other	38	12.0
	Iron sheets, or tiles	278	88.0
Major construction material of the house external wall	Unburnt bricks, mud and poles, thatch/straw, timber, stone, burnt bricks with mud, other	173	54.7
	Burnt bricks with cement, or cement blocks	143	45.3
Main source of income	Trading, commerce, selling (e.g., wholesalers, retailers, petty traders, etc.)	50	15.8
	Agriculture, livestock, forestry, fisheries (e.g., subsistence farmers, market vendors, etc.)	121	38.3
	Craft/creative workers (e.g., tailor, hairdresser, building, wood trades, metal and machinery)	112	35.4
	Transport industry (taxi, tri-cycles, etc.)	25	7.9
	Casual or wage labor (construction workers, farm laborers, etc.)	8	2.5
Second source of income	Trading, commerce, selling (e.g., wholesalers, retailers, petty traders, etc.)	316	100.0

### Basic knowledge about malaria

Basic knowledge about malaria can be found under Table 3. All the respondents had heard about malaria and 76.9% knew the mosquito as the malaria vector. Majority of respondents (69.9%) also knew that mosquito bites can transmit malaria. However, other routes of disease transmission chosen included drinking contaminated water (3.5%), and eating contaminated food (3.5%). Majority of the respondents (60.1%) knew untreated malaria could be fatal.

**Table 3:** Basic knowledge about malaria.

Variable	Categories	Frequency	Percent (%)
Have you ever heard about malaria?	Yes	316	100
	No	0	0
Which vector can transmit malaria to humans?	Mosquito	243	76.9
	Fly	50	15.8
	I don’t Know	23	7.3
Malaria can be transmitted to humans by?	Drinking contaminated water	11	3.5
	Eating contaminated food	11	3.5
	Bite of mosquito infected with malaria	221	69.9
	Coming into close contact with a malaria patient	73	23.1
Do you think malaria can kill you if its untreated?	Yes	190	60.1
	No	46	14.6
	I don’t Know	80	25.3
What do you think are the most common signs and symptoms of malaria infection?	High temperature/Fever	94	29.7
	Loss of energy	3	0.9
	Vomiting	43	13.6
	Sweating	1	0.3
	Headache	134	42.4
	Body pains	33	10.4
	Itching	8	2.5
Which of these are ways to prevent and control malaria?	Sleeping in bed nets	145	45.9
	Spraying insecticide	166	52.5
	I don’t Know	5	1.6
When do malaria mosquitoes’ feed?	Day time	23	7.3
	Night time	184	58.2
	Both day and night time	109	34.5
What personal protection measures do you use to guard against malaria?	Use repellents	35	11.1
	Use mosquito coil	186	58.9
	Close windows and doors	23	7.3
	Use mosquito nets	72	22.8
Does this household have bed nets?	Yes	131	41.5
	No	185	58.5
If yes, who owns the available nets in this household?	Father	8	2.5
	Mother	134	42.4
	Others	174	55.1
Are all these bed nets being used?	Yes	72	22.8
	No	244	77.2
Reason for net not being used	Heat	244	77.2
	Others	72	22.8

Headache (42.4%), high temperature/ fever (29.7%), and vomiting (13.6%), were the most common signs and symptoms indicated for malaria infection. The least signs and symptoms indicated for malaria infection were loss of energy (0.9%) and sweating (0.3%). Use of insecticide spray (52.5%) was indicated as the most common way to control and prevent malaria. Majority of the respondents (58.3%) were aware mosquitoes feed at night. The most common personal protection measure to guard against malaria (58.9%) was the use of mosquito coils. Majority of the households (58.5%) did not have bed nets and where they were available the ownership was by others (55.1%) but not the father or mother. Only a few bed nets (22.8%) were in use because of heat (77.2%).

### Sources of information about malaria

**Fig 2:**
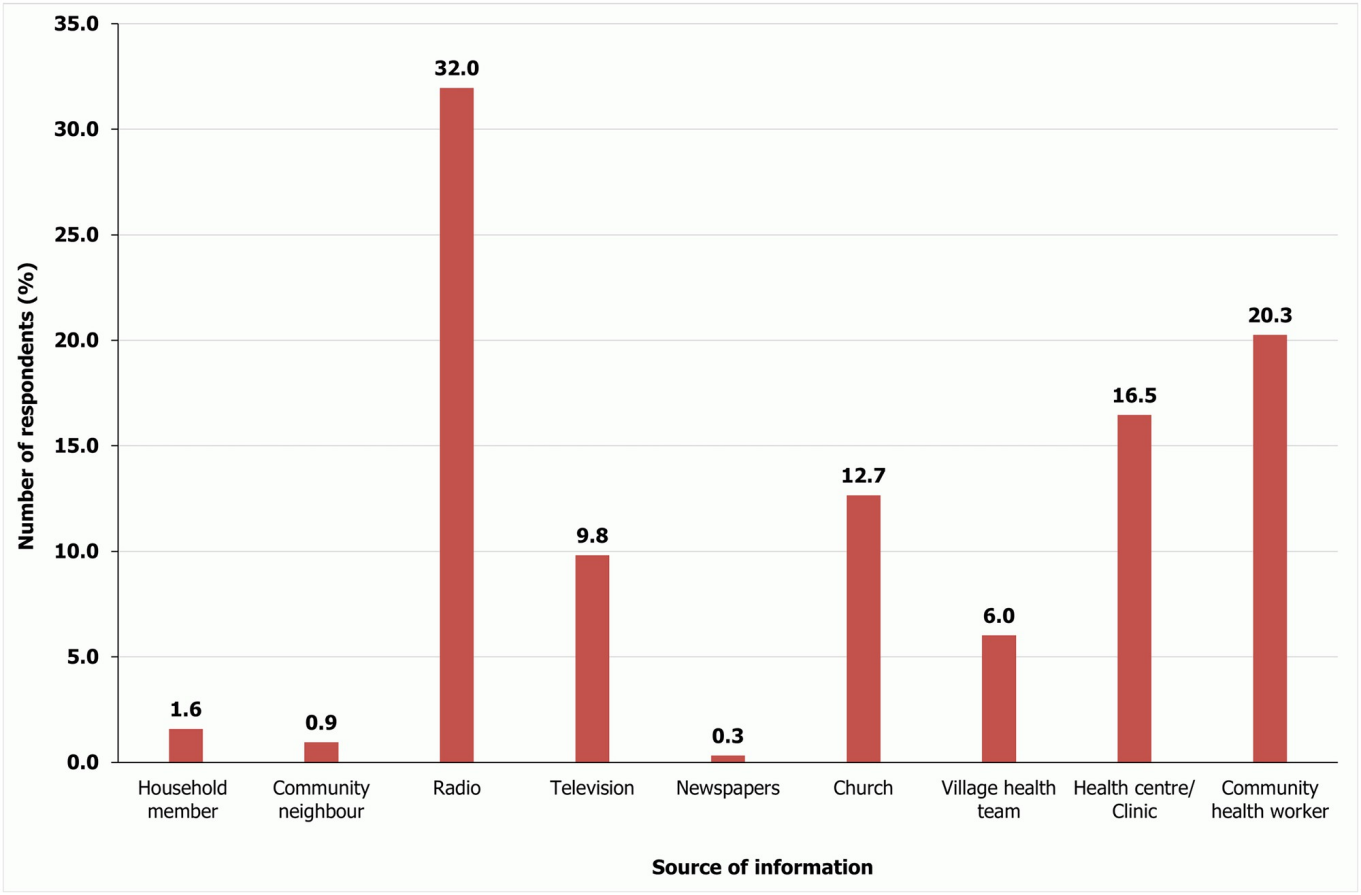
Sources from which information about malaria had been heard or received by respondents.

All the respondents had heard or received malaria-related information. The main information sources were radio (32.0%), a community health worker (20.3%) or a health center/clinic (16.5%) (Fig 2). Malaria-related information from newspapers (0.3%), a neighbor in the village (0.9%) and family member at home (1.65%) were the least.

### Treatment seeking behaviors

Most respondents (65.8%) reported a case of malaria in the household in the last six months (Table 4). The preferred choice of treatment seeking place (50.6%) was the health center/clinic, followed by a traditional healer (26.9%). Seeking treatment from a community health worker was the least (3.5%) option. All respondents indicated they would seek treatment within 24 hours. Most respondents (97.8%) indicated they did not have adequate information about malaria and wanted mainly information about prevention (42.7%), preferring the radio (40.5%) and the church (34.2%) as the primary sources (Table 4).

**Table 4:** Treatment seeking behaviors.

Variable	Categories	Frequency	Percent (%)
Have you or any member of the household suffered from malaria in the last six months?	Yes	208	65.8
	No	108	34.2
If you or a member of the household were to present with signs and symptoms of malaria, where would you seek treatment?	Health center/clinic	160	50.6
	Community health worker	11	3.5
	Traditional healer	85	26.9
	Drug shop /pharmacy	60	19.0
How soon after suspecting malaria would you seek treatment?	One day (within 24 hours)	316	100.0
Do you think you have enough information about malaria?	Yes	7	2.2
	No	309	97.8
If no, what information would you like to get about malaria?	Information on treatment	87	27.5
	Information on control	63	19.9
	Information on prevention	135	42.7
	Signs and symptoms	31	9.8
How would you like this information communicated to?	Family member (at home)	2	0.6
	Neighbor (in the village)	3	0.9
	Radio	128	40.5
	Television	1	0.3
	Church	108	34.2
	Health center/clinic	28	8.9
	Community health worker	25	7.9
	Drug shop /drug hawker	21	6.6

### Attitudes toward malaria

Majority of the respondents (60.1%) strongly agreed that malaria is a serious and life-threatening disease and all also strongly agreed to seek for advice or treatment when they get malaria (Table 5). Majority (88.9%) strongly disagreed that malaria can be transmitted like the common cold and almost half (49.4%) strongly disagreed that only pregnant women and children were at risk of getting malaria. Majority (50.6%) disagreed that recovery from malaria occurs spontaneously with no treatment and 55.1% also disagreed to avoiding people with malaria. Most respondents (50.3%) agreed anyone can get malaria, most (52.5%) also agreed that they can self-treat by anti-malaria drugs purchased from a pharmacy/drug store and most (81.6%) strongly agreed to take the drugs only after checking they had not expired. However, 37.0% (strongly disagree) and 33.5% (disagree) saw no danger with incomplete medication.

**Table 5:** Attitudes toward malaria.

	Frequency in attitude n (%)			
	Strongly Disagree	Disagree	Agree	Strongly agree
I think that malaria is a serious and life-threatening disease	6 (1.9)	17 (5.4)	103 (32.6)	190 (60.1)
Malaria can be transmitted from one person to another like the common cold	281 (88.9)	355 (11.1)	0 (0.0)	0 (0.0)
I think the best way to prevent myself getting malaria is to avoid getting mosquito bites cold	32 (10.1)	50 (15.8)	114 (36.1)	120 (38.0)
I am sure that anyone can get malaria	0.0	0.0	159 (50.3)	157 (49.7)
I believe sleeping under a mosquito net during the night is one way to prevent myself getting malaria	0.0	0.0	138 (43.7)	178 (56.3)
I am sure that I can treat myself if I get malaria	89 (28.2)	85 (26.9)	72 (22.8)	70 (22.2)
In my opinion, only children and pregnant women are at risk of malaria	156 (49.4)	137 (43.4)	18 (5.7)	5 (1.6)
I think that one can recover spontaneously from malaria without any treatment	138 (43.7)	160 (50.6)	12 (3.8)	6 (1.9)
If someone has got malaria, people should avoid having close contact with him/her	142 (44.9)	174 (55.1)	0 (0.0)	0 (0.0)
There is greater risk of getting malaria if I work and sleep overnight in the farm or forest	24 (7.6)	81 (25.6)	119 (37.7)	92 (29.1)
I think that it is dangerous when malaria medicine is not taken completely	118 (37.3)	106 (33.5)	60 (19.0)	32 (10.1)
I can buy anti-malaria drugs from the drug shop/pharmacy to treat myself when I get malaria	6 (1.9)	27 (8.5)	166 (52.5)	117 (37.0)
I think that I should go to the health center/clinic to have my blood tested as soon as I suspect that I have suffered from malaria	24 (7.6)	109 (34.5)	156 (49.4)	27 (8.5)
I will seek for advice or treatment when I get malaria	0 (0.0)	0 (0.0)	0 (0.0)	316 (100.0)
In my opinion, it is very important to check for an expiry date of the drug before taking it	0 (0.0)	0 (0.0)	58 (18.4)	258 (81.6)

Almost half (49.4%) agreed on having a blood test done at the health center on suspicion of having malaria and 28.2% strongly disagreed they can give themselves self- treatment. On prevention from getting malaria, most (56.3%) strongly agreed sleeping under a mosquito net at night can prevent them from getting malaria and 38.0% strongly agreed on avoiding mosquito bites. There was agreement (37.7%) and strong agreement (29.1%) of greater risk of malaria infection from working or sleeping overnight on the farm or in the forest.

### Practices toward malaria prevention

**Fig 3:**
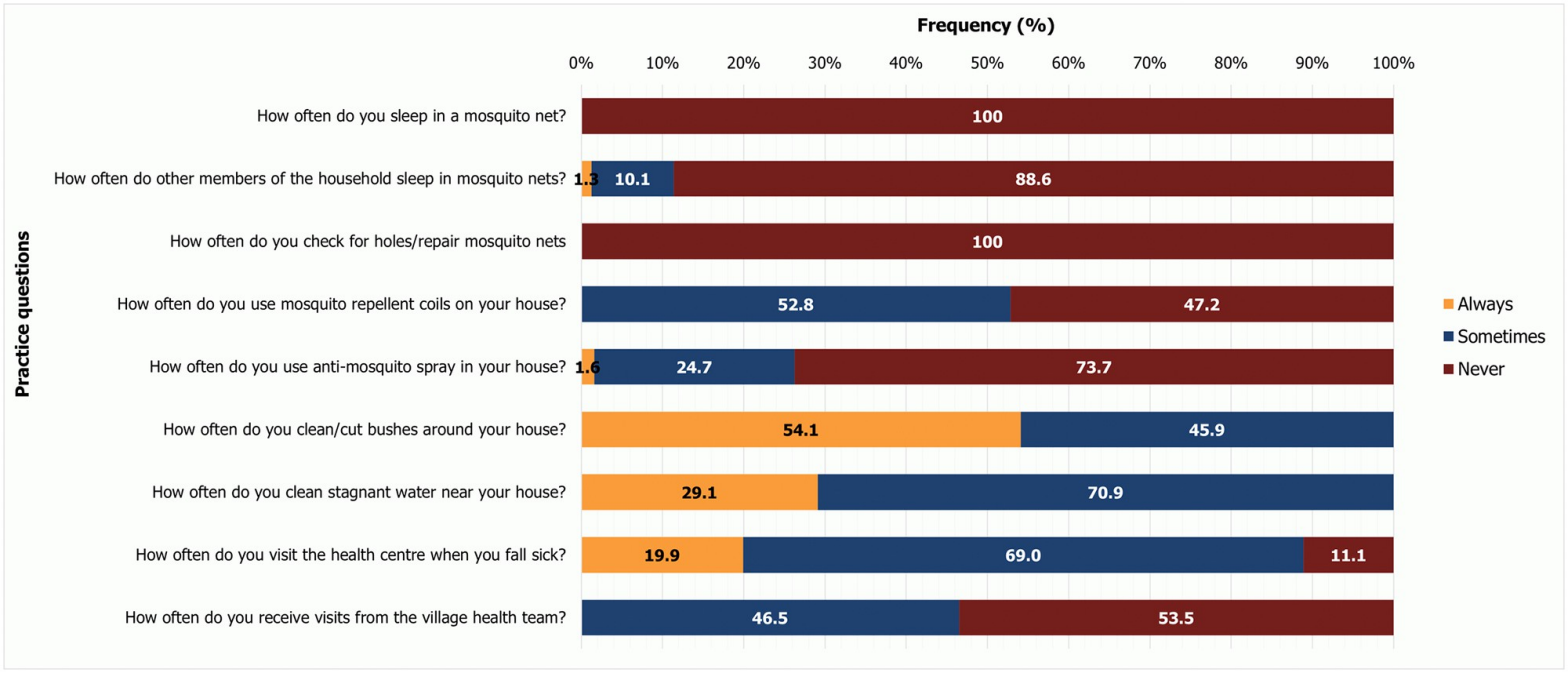
Practices toward malaria prevention.

All respondents reported that they never slept in mosquito nets and never checked or repaired holes in the nets (Fig 3). Also, only 10.1% of the households sometimes used mosquito nets. For prevention of mosquito bites, use of mosquito repellent coils was always used 52.8% of the time as compared with anti-mosquito sprays (24.7%). Draining stagnant water (29.1%) and clearing/cutting bushes (54.1%) made up the two main parts of the environmental management activities used to control the malaria vectors around the household.

### Overall KAP scores of the respondents and its association with of malaria control and prevention

According to the results of the knowledge score assessment, 85.4% of the respondents had good knowledge about malaria and nearly half (49.4%) had a positive attitude toward malaria in terms of its seriousness or threat, prevention, and control. Also, the overall practice score for malaria prevention and control among the respondents showed 40.5% had good practices.

### Associations of KAP scores of the respondents with their socio-demographic status

From the Chi-square tests (Tables A to C in S1 Table), none of the selected socio-demographic factors was associated with the knowledge score of malaria (all ps > 0.05). Chi-square analysis only showed significant associations for gender and attitude scores (p = 0.033), and educational status and practice scores (p = 0.023).

Tables D-F in S1 Table show the results of the binary logistic regression analyses. Respondents aged 51–60 years were 80% less likely to have good knowledge score [OR = 0.20 (95%CI: 0.04–0.95)] compared those aged 15–20 years. Respondents with complete basic school education were 67% less likely to have good knowledge score [OR = 0.33 (95%CI: 0.12–0.94)] compared to those with no formal schooling. The odds of positive attitude regarding malaria were 39% [OR = 0.61 (95%CI: 0.38–0.98)] lower among males compared to females (Table E in S1 Table). Compared to respondents with no formal schooling, those with incomplete basic school education level [OR = 0.30 (95%CI:0.12–0.77)] were 70% less likely to practice good malaria prevention (Table F in S1 Table).

## Discussion

Complex personal and societal factors that affect people’s behavior are linked to initiatives that seek to prevent and control malaria and lessen the burden that comes with it. These factors do, however, vary between nations and within communities. These highlight the importance of understanding local malaria risk factors. The current study therefore aimed to evaluate knowledge of malaria, attitudes toward the disease, adoption of control and prevention practices, and care-seeking behaviors in 13 communities across the Eastern Region of Ghana.

The majority of respondents were in the 41–50 years age range, which does not correspond to the Eastern Region’s current population proportion [31]. Due to the mainly rural nature of the communities used in the Eastern Region, about one-fifth of the respondents had no formal education. Our findings (38.3%) support agriculture as the primary source of income (32.0%) in the nation [31].

Results of the knowledge score assessment showed that 85.4% of the respondents had good knowledge about malaria. Thus, there was a high level of community awareness of malaria transmission, signs and symptoms, and treatment. This observation is comparable with the other studies in Swaziland [37], Northwest Ethiopia [22], Cameroon [17, 38], and North-western Tanzania [39]. However, the findings of this study are higher than the ones reported in Nigeria [23] and Southern Ethiopia [21, 40]. Although comparable to what other studies have found, the high level of knowledge may in part be due to health education campaigns [26]. A few respondents in this study, however, had knowledge gaps and included drinking contaminated water (3.5%), and eating contaminated food (3.5%) as ways that malaria is spread. It is very surprising that some respondents in this study and others conducted in malaria-endemic nations linked malaria to drinking contaminated water or other incorrect causes. In similar studies conducted in Zimbabwe [41] and Uganda [42], higher percentage of respondents provided the same answers. Studies in rural West African communities also reported comparable responses [43, 44].

How individual community members seek malaria treatment is crucial to the success of mortality prevention of the disease [45, 46]. Just over half (50.6%) of respondents indicated that health facilities were the best place to seek treatment for malaria although all respondents indicated they would seek treatment within 24 hours after suspecting malaria. Seeking treatment within 24 hours is extremely important especially in children under 5 years because they have little or no immunity and thus more likely to develop severe malaria [47]. Moreover, since malaria symptoms are generally non-specific and uncomplicated falciparum malaria can quickly progress to severe forms of the illness which can almost always lead to fatal outcomes without treatment, early diagnosis and prompt, effective treatment within 24–48 hours of the onset of malaria symptoms should be ensured [48]. In addition, since individual immunity can vary greatly, even in areas with moderate to high transmission intensity, the early treatment seeking behavior as indicated by all respondents is highly encouraging. It is also important to note that almost all the respondents (97.8%) wanted more information about prevention. This information was to come preferably from radio, which most respondent have access to on their phones, or from church, as majority of the respondents are Christians [31] and within the Ghanaian context, religion strongly influences how people live their daily lives [49]. Radio can be used to effectively spread malaria education because it is frequently stated that people access health information on radio [50, 51] as also revealed in this study.

In the present study, majority of the respondents (60.1%) strongly agreed that malaria is a serious and life-threatening disease which is lower compared with a study carried out in Ethiopia [19]. Despite this, all the respondents strongly agreed to seek for advice or treatment when they contract malaria, and almost half agreed on having a blood test done at the health center on suspicion of having malaria although majority (52.5%) also agreed to self-medication. Studies in Ghana [29, 52] and countries where malaria is endemic, Uganda [53], Southern Sudan [54], Tanzania [55] and Kenya [56], have noted the use of drugs purchased from drug stores and general stores for self-treatment. Even more intriguingly, studies conducted in Nigeria [57] and Ghana [52] revealed that self-medication with herbal preparations is regarded as the primary method of treating malaria. Therefore, it is clear that taking self-medication causes patients to put off getting the proper medical attention, which could worsen malaria outcomes. Self-medication may also be the cause of the observed noncompliance with national malaria treatment recommendations, which affects treatment outcomes and aids in the emergence of drug resistance. What was also a concern was the fact that 37.0% (strongly disagree) and 33.5% (disagree) saw no danger with incomplete medication which can result in treatment failure when complete doses are admitted later [58, 59].

On prevention from getting malaria, although most (56.3%) strongly agreed sleeping under a mosquito net at night can prevent them from getting malaria and 38.0% strongly agreed on avoiding mosquito bites, in practice mosquito net usage was largely absent. Only 10.1% of households sometimes used nets and all the respondents never used them. Low net usage (15%) was also reported in a study in Ghana [29]. This practice starkly disagrees with net usage in studies done in Ghana [27], and in other countries such as Cameroon [17, 18], Tanzania [39], Ethiopia [19, 21], Guinea [50], and Nigeria [24]. The main reason given by majority of respondents (77.2%) for the non-usage of the nets in this study was heat. This agrees with results of the other studies in Ghana [27, 29] and a study in Cameroon [17]. To increase the use of bed nets, continuous behavior change communication should be carried out in the districts [60]. In this current study, respondents preferred use of mosquito coils (52.8%) for prevention of mosquito bites in agreement with other studies in Ghana [27, 28]. Clearing of bushes against mosquito bites was also high (54.1%) among the households. Use of multiple mosquito control and preventive methods have been observed in other studies [18, 27, 28].

In this study, despite the high good knowledge score (85.4%) among the respondents, this was not reflected in their attitude (50.6% negative attitude) and practice (59.5% poor practice) scores toward malaria control and prevention. The negative attitude scores are higher than those reported from studies in Ethiopia [19]. The practice scores in this study are also lower than those of studies in Ethiopia [19, 40] and Sri Lanka [61] but higher than that of the study in Lao PDR [62]. Studies on bed net use and malaria prevention have found instances where participants’ increased knowledge did not result in better use of bed nets thus raising doubt on behavioral theories that presuppose a linear relationship between comprehension and application of malaria prevention strategies. Knowledge scores may be affected by differences in communities’ demographic, socioeconomic, educational, and cultural characteristics and by the lack of, access to, or accuracy of information about malaria [26–28, 52]. This present study revealed that female gender was the explanatory variable which influenced positive attitude to malaria control and prevention although the majority of respondents were males. This disagrees with studies carried out in rural Ghana [26] and Bangladesh [51]. Given the patriarchal nature in the Ghanaian society [49] this is surprising. This study’s findings indicated that respondents’ education status in general did not significantly influence good practice of malaria prevention and control efforts. This might be due to the fact that about a third of the respondents had incomplete educational status. The degree of education, however, has shown a significant influence on populations’ malaria-related KAP in many parts of the world [8, 17, 19, 20, 51].

## Conclusions

Overall score for respondents’ knowledge of malaria was good. However, attitudes and levels of practice regarding malaria did not match the knowledge score. Bednet use was almost non-existent. Health education, preferably on radio and at churches, aimed at prevention and control of the disease through changes in attitude and practice is urgently required.

## Supporting information

S1 File Study questionnaire. (PDF)

S2 File Malaria KAP dataset. (SAV)

S1 Table Tables presenting the results of Chi-square tests and binary logistic regression analyses for the associations of KAP scores of the respondents with their socio-demographic status. (DOCX)
