# Supplementary material for: Knowledge, attitudes and practices regarding malaria prevention and control in communities in the Eastern Region, Ghana, 2020
Source: PLoS One. 2023 Aug 30;18(8):e0290822. doi: 10.1371/journal.pone.0290822 (PMC10468076; doi:10.1371/journal.pone.0290822)
Supplement: S1 File — (PDF) [file pone.0290822.s001.pdf]

Survey Questionnaire- English

Assessment of Knowledge, Attitudes and Practices Regarding Malaria Prevention and Control

Date:______________________

Interviewer: ___________________

Entry ___________________ Number: ___________________
(To be filled by the data entry person before entering the data of this questionnaire)

1. Demographics

1. Name of village: _____________________________________

2. Gender of interviewee

Female	☐	(1)
Male	☐	(2)

3. How old are you (years)? (Tick only one box)

15 - 20	☐	(1)
21 – 30	☐	(2)
31 – 40	☐	(3)
41 – 50	☐	(4)
51 – 60	☐	(5)
Above 60	☐	(6)

4. What is the highest level of education that you have achieved? (Tick only one box)

No formal schooling	☐	(1)
Incomplete basic school	☐	(2)
Complete basic school (B1-B6/JHS)	☐	(3)
Incomplete secondary school (SHS)	☐	(4)
Complete secondary school (SHS)	☐	(5)
Post-secondary e.g., certificate, diploma, degree	☐	(6)
Degree and above	☐	(7)

5. How many people live in your household including you? (Includes biological children or other dependents, tick only one box)

Six or more	☐	(1)
Four or five	☐	(2)
Three	☐	(3)
Two	☐	(4)
One	☐	(5)

6. What is your relationship to the head of the household? (Tick only one box)

I am head of household	☐	(1)
Spouse/partner	☐	(2)
Son/daughter	☐	(3)
Grandchild	☐	(4)
Parent	☐	(5)
Brother /sister	☐	(6)
Not related	☐	(7)
Other	☐	(8)

If "other" describe:

____

7. What is the highest grade that the female head/spouse completed? (Tick only one box)

No female head/spouse	☐	(1)
Basic 1-6 or less, or none	☐	(2)
Basic 7-9 (JHS)	☐	(3)
SHS	☐	(4)
Higher than SHS	☐	(5)

8. What is the major construction material of the roof? (Tick only one box)

Thatch, straw, or other ☐ (1)
Iron sheets, or tiles ☐ (2)

9. What is the major construction material of the external wall? (Tick only one box)

Un-burnt bricks, mud and poles, thatch/straw, timber, stone, burnt bricks with mud, other ☐ (1)

Burnt bricks with cement, or cement blocks ☐ (2)

10. What is the main source of lighting in your dwelling? (Tick only one box)

Paraffin lantern ☐ (1)
Solar light ☐ (2)
Electricity (ECG, generator) ☐ (3)

11. What is the type of toilet that is mainly used in your household? (Tick only one box)

Bush (none) ☐ (1)
Covered pit latrine (private or shared), VIP latrine (private or shared), uncovered pit latrine, flush toilet (private or shared), or other ☐ (2)

12. Does any member of your household own electronic equipment (e.g., TV, radio, cassette, etc.) at present? (Tick only one box)

No ☐ (0)
Yes ☐ (1)

13. What is your main source of income? (Tick only one)

Formal employment (e.g., nurse, teacher, secretary, accountant, etc.) ☐ (1)
Trading, commerce, selling (e.g., wholesalers, retailers, petty traders, etc.) ☐ (2)
Agriculture, livestock, forestry, fisheries (e.g., subsistence farmers, market vendors, etc.) ☐ (3)
Craft/creative workers (e.g. tailor, hairdresser, building, wood trades, metal and machinery) ☐ (4)
Transport industry (trotro, taxi, tricycles, etc.) ☐ (5)
Casual or wage labour (construction workers, farm labourers, etc.) ☐ (6)
Support from friends/family (husband/wife, students, remittance from friends/family) ☐ (7)
Support from institutions (government, NGO payments, pensions, etc.) ☐ (8)

14. What is your second source of income? (Tick only one)

Formal employment (e.g., nurse, teacher, secretary, accountant, etc.) ☐ (1)

Trading, commerce, selling (e.g., wholesalers, retailers, petty traders, etc.) ☐ (2)

Agriculture, livestock, forestry, fisheries (e.g., subsistence farmers, market vendors, etc.) ☐ (3)

Craft/creative workers (e.g. tailor, hairdresser, building, wood trades, metal and machinery) ☐ (4)

Transport industry (trotro, taxi, bicycles, etc.) ☐ (5)

Casual or wage labour (construction workers, farm labourers, etc.) ☐ (6)

Support from friends/family (husband/wife, students, remittance from friends/family) ☐ (7)

Support from institutions (government, NGO payments, pensions, etc.) ☐ (8)

None ☐ (9)

2. Basic Knowledge about Malaria

15. Have you ever heard about malaria?

Yes	☐	(1)
No	☐	(2)
I don't Know	☐	(3)

16. Which vector can transmit malaria to humans? (Tick one only)

Rat	☐	(1)
Dog	☐	(2)
Mosquito	☐	(3)
Fly	☐	(4)
Cockroach	☐	(5)
I don't Know	☐	(6)

17. Malaria can be transmitted to humans by?

Drinking contaminated water	☐	(1)
Eating contaminated food	☐	(2)
Eating a lot of mangoes	☐	(3)
Bite of mosquito infected with Malaria	☐	(4)
Coming into close contact with a Malaria patient	☐	(5)

18. Do you think malaria can kill you if its untreated?

Yes	☐	(1)
No	☐	(2)
I don't Know	☐	(3)

19. What do you think are the most common signs and symptoms of malaria infection? (Tick all that apply)

High temperature/Fever	☐	(1)
Loss of energy	☐	(2)
Vomiting	☐	(3)
Sweating	☐	(4)
Headache	☐	(5)
Body pains	☐	(6)
Itching	☐	(7)
Loss of appetite	☐	(8)
Chills	☐	(9)
Dizziness	☐	(10)

I don't Know	☐	(11)
Other	☐	(12)

If "other" describe:

---

20. Which of these are ways to prevent and control malaria? (Tick all that apply)

Sleeping in bed nets	☐	(1)
Wearing long sleeved clothes	☐	(2)
Making fire and smoke	☐	(3)
Spraying insecticide	☐	(4)
Trimming bushes around the house pain	☐	(5)
Cleaning dark corners in the house	☐	(6)
I don't Know	☐	(7)

21. When do malaria mosquitoes feed? (Tick only one)

Daytime	☐	(1)
Night time	☐	(2)
Both day and night time	☐	(3)
I don't Know	☐	(4)

22. What personal protection measures do you use to guard against malaria? (Tick all that apply)

Use repellents	☐	(1)
Use mosquito coil	☐	(2)
Use doom	☐	(3)
Burn cow dung/leaves	☐	(4)
Close windows and doors	☐	(5)
Gauze wire in windows	☐	(6)
Use mosquito nets	☐	(7)
Do nothing	☐	(8)
Others (specify)	☐	(9)

<u>Bed net ownership and use</u>

23. Does this household have bed nets?

Yes	☐	(1)
No	☐	(2)

24. If yes, who owns the available nets in this household? (Tick as many)

Father	☐	(1)
Mother	☐	(2)
Children over five years	☐	(3)
Children under five years	☐	(4)
Others (Specify)	☐	(5)

25. Are all these bed nets being used?

Yes	☐	(1)
No	☐	(2)
Don't know	☐	(3)

26. If no to question 25, why? _________________

3. Sources of Information about Malaria

27. Have you ever heard or received any information related malaria? (Tick only one)

Yes	☐	(1)
No	☐	(2)
I don't Know	☐	(3)

28. If yes, From which sources have you heard or received information about malaria? (Tick all that apply)

Family member (at home)	☐	(1)
Neighbour (in the village)	☐	(2)
Radio	☐	(3)
Television	☐	(4)
Newspapers	☐	(5)
Posters/pamphlets	☐	(6)
School	☐	(7)
Church	☐	(8)
LC Chairman/Health Secretary	☐	(9)
Village Health Team	☐	(10)
Health centre/clinic	☐	(11)
Community health worker	☐	(12)
Health centre/clinic	☐	(13)
Drug shop /drug hawker	☐	(14)
Other	☐	(15)

If "other" describe:

___

4. Treatment Seeking Behaviours

29. Have you or any member of the household suffered from malaria in the last six months (Tick only one)

Yes	☐	(1)
No	☐	(2)
I don't Know	☐	(3)

30. If you or a member of the household were to present with signs and symptoms of malaria, where would you seek treatment

Health centre/clinic	☐	(1)
Community health worker	☐	(2)
Traditional healer	☐	(3)
Drug shop /pharmacy	☐	(4)
Look for local herbs	☐	(5)
No where	☐	(6)
I don't Know	☐	(7)
Other	☐	(8)

If "other" describe: ______________________________

31. How soon after suspecting malaria would you seek treatment?

One day(within 24 hours)	☐	(1)
2-3 days	☐	(2)
4-6 days	☐	(3)
7 days or more	☐	(4)
I don't Know	☐	(5)

32. If you do not seek treatment immediately (within 24 hours), what would you do? ____

33. Do you think you have enough information about malaria? (Tick only one)

Yes	☐	(1)
No	☐	(2)
I don't Know	☐	(3)

34. If No, what information would you like to get about malaria?

Information on treatment	☐	(1)
Information on control	☐	(2)
Information on prevention	☐	(3)
Signs and symptoms	☐	(4)
Nature of the disease	☐	(5)
Any information	☐	(6)
I don't Know	☐	(7)
Other	☐	(8)

If "other" describe:

---

35. How would you like this information communicated to? (Tick all that apply)

Family member (at home)	☐	(1)
Neighbour (in the village)	☐	(2)
Radio	☐	(3)
Television	☐	(4)
Newspapers	☐	(5)
Posters/pamphlets	☐	(6)
School	☐	(7)
Church	☐	(8)
LC Chairman/Health Secretary	☐	(9)
Village Health Team	☐	(10)
Health centre/clinic	☐	(11)
Community health worker	☐	(12)
Health centre/clinic	☐	(13)
Drug shop /drug hawker	☐	(14)
Other	☐	(15)

If "other" describe:

5. Attitudes Towards Malaria

	Strongly Disagree	Disagree	Agree	Strongly Agree
36. I think that malaria is a serious and life-threatening disease	[1] ☐	[2] ☐	[3] ☐	[4] ☐
37. Malaria can be transmitted from one person to another like the common cold	[1] ☐	[2] ☐	[3] ☐	[4] ☐
38. I am sure that anyone can get malaria	[1] ☐	[2] ☐	[3] ☐	[4] ☐
39. I believe sleeping under a mosquito net during the night is one way to prevent myself getting malaria	[1] ☐	[2] ☐	[3] ☐	[4] ☐
40. I am sure that I can treat myself if I get malaria	[1] ☐	[2] ☐	[3] ☐	[4] ☐
41. In my opinion, only children and pregnant women are at risk of malaria	[1] ☐	[2] ☐	[3] ☐	[4] ☐
42. I think that one can recover spontaneously from malaria without any treatment	[1] ☐	[2] ☐	[3] ☐	[4] ☐
43. If someone has got malaria, people should avoid having close contact with him/her	[1] ☐	[2] ☐	[3] ☐	[4] ☐
44. I might be at a greater risk of getting malaria if I work and sleep overnight in the garden or forest	[1] ☐	[2] ☐	[3] ☐	[4] ☐
45. I think that it is dangerous when malaria medicine is not taken completely	[1] ☐	[2] ☐	[3] ☐	[4] ☐
46. I can buy anti-malaria drugs from the drug shop/pharmacy to treat myself when I get malaria	[1] ☐	[2] ☐	[3] ☐	[4] ☐
47. I think that I should go to the health centre/clinic to have my blood tested as soon as I suspect that I have suffered from malaria	[1] ☐	[2] ☐	[3] ☐	[4] ☐
48. I will seek for advice or treatment when I get malaria	[1] ☐	[2] ☐	[3] ☐	[4] ☐
49. In my opinion, it is very important to check for an expiry date of the drug before taking it	[1] ☐	[2] ☐	[3] ☐	[4] ☐

6. Practices Towards Malaria Prevention

	Always	Sometimes	Never
50. How often do you sleep in a mosquito net?	[1] ☐	[2] ☐	[3] ☐
51. How often do other members of the household sleep in mosquito nets?	[1] ☐	[2] ☐	[3] ☐
52. How often do you check for holes/repair mosquito nets	[1] ☐	[2] ☐	[3] ☐
53. How often do you use mosquito repellent coils on your house?	[1] ☐	[2] ☐	[3] ☐
54. How often do you use anti-mosquito spray in your house?	[1] ☐	[2] ☐	[3] ☐
55. How often do you clean/cut bushes around your house?	[1] ☐	[2] ☐	[3] ☐
56. How often do you clean stagnant water near your house	[1] ☐	[2] ☐	[3] ☐
57. How often do you visit the health centre when you fall sick?	[1] ☐	[2] ☐	[3] ☐
58. How often to you receive visits from the village health team?	[1] ☐	[2] ☐	[3] ☐
