# Supplementary material for: Knowledge, attitudes and practices regarding malaria prevention and control in communities in the Eastern Region, Ghana, 2020
Source: PLoS One. 2023 Aug 30;18(8):e0290822. doi: 10.1371/journal.pone.0290822 (PMC10468076; doi:10.1371/journal.pone.0290822)
Supplement: S1 Table — (DOCX) [file pone.0290822.s003.docx]

**Table A. Associations of knowledge score of the respondents with their socio-demographic status**

|  |  | **Total** | **Knowledge score,**  **n (%)** | |  |  |
| --- | --- | --- | --- | --- | --- | --- |
| **Variable** | **Categories** | **n (%)** | **Poor** | **Good** | **χ^2^** | **p-value** |
| **Gender of respondent** | Female | 125 (39.6) | 20 (43.5) | 105 (38.9) | 0.346 | 0.556 |
|  | Male | 191 (60.4) | 26 (56.5) | 165 (61.1) |  |  |
| **Age (years)** | 15-20 | 56 (17.7) | 6 (13.0) | 50 (18.5) | 6.642 | 0.249 |
|  | 21-30 | 74 (23.4) | 7 (15.2) | 67 24.8) |  |  |
|  | 31-40 | 49 (15.5) | 10 (21.7) | 39 (14.4) |  |  |
|  | 41-50 | 119 (37.7) | 19 (41.3) | 100 (37.0) |  |  |
|  | 51-60 | 14 (4.4) | 4 (8.7) | 10 (3.7) |  |  |
|  | Above 60 | 4 (1.3) | 0 (0.0) | 4 (1.5) |  |  |
| **Highest level of education** | No formal schooling | 62 (19.6) | 6 (13.0) | 56 (20.7) | 7.07 | 0.314 |
|  | Incomplete basic school | 38 (12.0) | 5 (10.9) | 33 (12.2) |  |  |
|  | Complete basic school | 79 (25.0) | 18 (39.1) | 61 (22.6) |  |  |
|  | Incomplete secondary school (SHS) | 33 (10.4) | 4 (8.7) | 29 (10.7) |  |  |
|  | Complete secondary school (SHS) | 32 (10.1) | 5 (10.9) | 27 (10.0) |  |  |
|  | Post-secondary e.g., certificate, diploma | 66 (20.9) | 8 (17.4) | 58 (21.5) |  |  |
|  | Degree and above | 6 (1.9) | 0 (0.0) | 6 (2.2) |  |  |
|  |  |  |  |  |  |  |

**Table B. Associations of attitude score of the respondents with their socio-demographic status**

|  | | **Total** | **Attitude score, n (%)** | |  |  |
| --- | --- | --- | --- | --- | --- | --- |
| **Variable** | **Categories** | **n (%)** | **Negative** | **Positive** | **χ^2^** | **p-value** |
| **Gender of respondent** | Female | 125 (39.6) | 54 (33.8) | 71 (45.5) | 4.157 | **0.033*** |
|  | Male | 191 (60.4) | 106 (66.3) | 85 (54.5) |  |  |
| **Age (years)** | 15-20 | 56 (17.7) | 27 (16.9) | 29 (18.6) | 10.631 | 0.059 |
|  | 21-30 | 74 (23.4) | 46 (28.7) | 28 (17.9) |  |  |
|  | 31-40 | 49 (15.5) | 18 (11.3) | 31 (19.9) |  |  |
|  | 41-50 | 119 (37.7) | 57 (35.6) | 62 (39.7) |  |  |
|  | 51-60 | 14 (4.4) | 10 (6.3) | 4 (2.6) |  |  |
|  | Above 60 | 4 (1.3) | 2 (1.3) | 2 (1.3) |  |  |
| **Highest level of education** | No formal schooling | 62 (19.6) | 30 (18.8) | 32 (20.5) | 6.92 | 0.328 |
|  | Incomplete basic school | 38 (12.0) | 22 (13.8) | 16 (10.3) |  |  |
|  | Complete basic school | 79 (25.0) | 36 (22.5) | 43 (27.6) |  |  |
|  | Incomplete secondary school (SHS) | 33 (10.4) | 20 (12.5) | 13 (8.3) |  |  |
|  | Complete secondary school (SHS) | 32 (10.1) | 13 (8.1) | 19 (12.2) |  |  |
|  | Post-secondary e.g., certificate, diploma | 66 (20.9) | 34 (21.3) | 32 (20.5) |  |  |
|  | Degree and above | 6 (1.9) | 5 (3.1) | 1 (0.6) |  |  |

*Significant at p<0.05

**Table C. Associations of practice score of the respondents with their socio-demographic status**

|  | | **Total** | **Practice score, n (%)** | |  |  |
| --- | --- | --- | --- | --- | --- | --- |
| **Variable** | **Categories** | **n (%)** | **Bad** | **Good** | **χ^2^** | **p-value** |
| **Gender of respondent** | Female | 125 (39.6) | 74 (39.4) | 51 (39.8) | 0.01 | 0.931 |
|  | Male | 191 (60.4) | 114 (60.6) | 77 (60.2) |  |  |
| **Age (years)** | 15-20 | 56 (17.7) | 31 (16.5) | 25 (19.5) | 3.87 | 0.586 |
|  | 21-30 | 74 (23.4) | 45 (23.9) | 29 (22.7) |  |  |
|  | 31-40 | 49 (15.5) | 31 (16.5) | 18 (14.4) |  |  |
|  | 41-50 | 119 (37.7) | 68 (36.2 | 51 (39.8) |  |  |
|  | 51-60 | 14 (4.4) | 9 (4.8) | 5 (3.9) |  |  |
|  | Above 60 | 4 (1.3) | 4 (2.1) | 0 (0.0) |  |  |
| **Highest level of education** | No formal schooling | 62 (19.6) | 35 (18.6) | 27 (21.1) | 14.65 | **0.023*** |
|  | Incomplete basic school | 38 (12.0) | 30 (16.0) | 8 (6.3) |  |  |
|  | Complete basic school | 79 (25.0) | 46 (24.5) | 33 (25.8) |  |  |
|  | Incomplete secondary school (SHS) | 33 (10.4) | 19 (10.1) | 14 (10.9) |  |  |
|  | Complete secondary school (SHS) | 32 (10.1) | 23 (12.2) | 9 (7.0) |  |  |
|  | Post-secondary e.g., certificate, diploma | 66 (20.9) | 34 (18.1) | 32 (25.0) |  |  |
|  | Degree and above | 6 (1.9) | 1 (0.5) | 5 (3.9) |  |  |

*Significant at p<0.05

**Table D. A logistic regression of the determinants related to knowledge of malaria among the study participants**

|  |  |  | **95% CI for OR** | |  |
| --- | --- | --- | --- | --- | --- |
| **Variable** | **Categories** | **OR** | **Lower** | **Upper** | **P** |
| **Gender of respondent** | Female | 1 |  |  |  |
|  | Male | 1.22 | 0.63 | 2.38 | 0.55 |
| **Age (years)** | 15-20 | 1 |  |  |  |
|  | 21-30 | 1.08 | 0.34 | 3.47 | 0.90 |
|  | 31-40 | 0.47 | 0.15 | 1.43 | 0.18 |
|  | 41-50 | 0.57 | 0.21 | 1.54 | 0.27 |
|  | 51-60 | 0.20 | 0.04 | 0.95 | **0.04*** |
|  | Above 60 | 113096846.80 | 0.00 |  | 1.00 |
| **Highest level of education** | No formal schooling | 1 |  |  |  |
|  | Incomplete basic school | 0.65 | 0.17 | 2.39 | 0.51 |
|  | Complete basic school | 0.33 | 0.12 | 0.94 | **0.04*** |
|  | Incomplete secondary school (SHS) | 0.81 | 0.20 | 3.26 | 0.77 |
|  | Complete secondary school (SHS) | 0.50 | 0.13 | 1.91 | 0.31 |
|  | Post-secondary e.g., certificate, diploma | 0.71 | 0.22 | 2.29 | 0.56 |
|  | Degree and above | 183145337.45 | 0.00 |  | 1.00 |
|  |  |  |  |  |  |

* Significant at p<0.05

1 = Reference.

**Table E. A logistic regression of the determinants related to attitude towards malaria among the study participants**

|  |  |  | **95% CI for OR** | |  |
| --- | --- | --- | --- | --- | --- |
| **Variable** | **Categories** | **OR** | **Lower** | **Upper** | **P** |
| **Gender of respondent** | Female | 1 |  |  |  |
|  | Male | 0.61 | 0.38 | 0.98 | **0.04*** |
| **Age (years)** | 15-20 | 1 |  |  |  |
|  | 21-30 | 0.55 | 0.27 | 1.13 | 0.10 |
|  | 31-40 | 1.67 | 0.75 | 3.72 | 0.21 |
|  | 41-50 | 1.10 | 0.57 | 2.13 | 0.78 |
|  | 51-60 | 0.40 | 0.11 | 1.49 | 0.17 |
|  | Above 60 | 0.86 | 0.10 | 7.01 | 0.89 |
| **Highest level of education** | No formal schooling | 1 |  |  |  |
|  | Incomplete basic school | 0.65 | 0.28 | 1.53 | 0.33 |
|  | Complete basic school | 0.99 | 0.49 | 1.99 | 0.97 |
|  | Incomplete secondary school (SHS) | 0.71 | 0.29 | 1.73 | 0.45 |
|  | Complete secondary school (SHS) | 1.37 | 0.56 | 3.38 | 0.49 |
|  | Post-secondary e.g., certificate, diploma | 0.76 | 0.36 | 1.59 | 0.47 |
|  | Degree and above | 0.15 | 0.02 | 1.44 | 0.10 |
|  |  |  |  |  |  |

* Significant at p<0.05

1 = Reference.

**Table F. A logistic regression of the determinants related to adoption of malaria control and prevention practices among the study participants**

|  |  |  | **95% CI for OR** | |  |
| --- | --- | --- | --- | --- | --- |
| **Variable** | **Categories** | **OR** | **Lower** | **Upper** | **P** |
| **Gender of respondent** | Female | 1 |  |  |  |
|  | Male | 1.06 | 0.65 | 1.71 | 0.83 |
| **Age (years)** | 15-20 | 1 |  |  |  |
|  | 21-30 | 0.75 | 0.36 | 1.55 | 0.44 |
|  | 31-40 | 0.67 | 0.30 | 1.51 | 0.34 |
|  | 41-50 | 0.79 | 0.40 | 1.56 | 0.50 |
|  | 51-60 | 0.61 | 0.17 | 2.18 | 0.45 |
|  | Above 60 | 0.00 | 0.00 |  | 1.00 |
| **Highest level of education** | No formal schooling | 1 |  |  |  |
|  | Incomplete basic school | 0.30 | 0.12 | 0.77 | **0.01*** |
|  | Complete basic school | 0.82 | 0.41 | 1.65 | 0.58 |
|  | Incomplete secondary school (SHS) | 0.91 | 0.38 | 2.19 | 0.83 |
|  | Complete secondary school (SHS) | 0.45 | 0.18 | 1.16 | 0.10 |
|  | Post-secondary e.g., certificate, diploma | 1.08 | 0.53 | 2.23 | 0.83 |
|  | Degree and above | 5.71 | 0.61 | 53.15 | 0.13 |
|  |  |  |  |  |  |

* Significant at p<0.05

1 = Reference.
